# Physiological status of broiler chickens with diets supplemented with milk thistle extract

**DOI:** 10.14202/vetworld.2021.1319-1323

**Published:** 2021-05-26

**Authors:** Olga Bagno, Sergey Shevchenko, Antonina Shevchenko, Oleg Prokhorov, Anna Shentseva, Grigory Vavin, Elena Ulrich

**Affiliations:** 1 Zootechnical Faculty, Kuzbass State Agricultural Academy, Russia; 2 Institute of Physics and Mathematics and Engineering and Technology, Gorno-Altai State University, Russia; 3 Kuzbass Clinical Hospital named after S.V. Belyaev, Russia; 4 Faculty of Technological Entrepreneurship, Kuzbass State Agricultural Academy, Russia

**Keywords:** blood biochemical composition, feed supplement, hemoglobin, milk thistle (*Silybum marianum*), red blood cells, white blood cells

## Abstract

**Background and Aim:**

In recent decades, the use of various feed supplements is the current trend in poultry farming, among which phytogenics serve as alternatives to feed antibiotics. This study aimed to examine the effect of feeding various doses of milk thistle extract (*Silybum marianum*) on the morphological and biochemical parameters of the blood in broiler chickens.

**Materials and Methods:**

Experiments were carried out in an industrial poultry farm on broiler chickens of the Hubbard ISA F15 cross for 40 days. One control group and five experimental groups of day-old chickens were formed. The number of birds in each group was 50. Broilers of all groups received complete feed, and the experimental groups received an additional milk thistle extract at doses of 0.1, 0.5, 1.0, 1.5, and 2.0 mg/kg of body weight. Milk thistle medicinal plant extract was obtained using water-ethanol extraction followed by low-temperature vacuum drying. For the assessment of blood analyses, samples were collected from the wing vein of six chickens per group. Using unified methods recommended by the International Federation of Clinical Chemistry, the content of red blood cells, hemoglobin, white blood cells, total protein, protein fractions, triglycerides, glucose, calcium, phosphorus, and the concentration of alanine aminotransferase and aspartate aminotransferase in the blood serum were determined.

**Results:**

It was found that the introduction of milk thistle extract into the diet of broiler chickens with the aforementioned doses increased the number of red blood cells, hemoglobin, white blood cells in the blood, as well as a decrease in the level of albumin and an increase in the content of γ-globulins in its serum.

**Conclusion:**

The authors assume that the introduction of milk thistle extract into a complete feed for broiler chickens increased the anabolic processes in their bodies, accompanied by increased use of proteins of the albumin fraction as the main material for organogenesis.

## Introduction

When methods aimed at improving productive efficiency were used in industrial poultry farming, the load on all functional systems of the birds’ bodies inevitably increases. Intensification of feeding and impairment of welfare decrease birds’ resistance, causing the percentage of morbidity to increase, safety issues to arise, and productivity to decrease. The liver is the organ that primarily reacts to the influence of the above factors. To prevent the negative impact of technological factors in the poultry industry, various feed supplements are used. The introduction of functional feed supplements in the diet of poultry improves regenerative processes in the birds’ bodies as a whole, particularly the liver, to reduce the negative consequences of intensive cultivation technologies and obtain competitive high-quality products [1].

In recent years, technologies based on replacing synthetic feed antibiotics with alternative agents, such as biologically active substances extracted from medicinal plants with various properties, have become a global interest [2-4]. The natural origin of phytogenic feed supplements determines their environmental safety [5,6]. One of the medicinal plants currently widely used in medicine is milk thistle (*Silybum marianum*). The effects of using milk thistle are determined by the main biologically active compounds in its fruits. Thus, silybin acts as a stabilizer of permeability of cell membranes, protecting hepatocytes from toxic substances, and is used as a hepatorenal protective agent in poultry. Silymarin can protect the liver, brain, heart, and other vital organs from oxidative damage through its ability to prevent lipid peroxidation and replenish low glutathione levels [7-10].

At present, the use of milk thistle extract in poultry remains poorly understood. In particular, there are no data on the morphobiochemical composition in the blood of poultry with the introduction of various doses of extracts produced using water-ethanol extraction.

This study aimed to determine the physiological status of broiler chickens when fed with various doses of milk thistle extract.

## Materials and Methods

### Ethical approval

All studies were approved by the ethical committee of the Kuzbass State Agricultural Academy (protocol No. 3 dated 13.02.2018).

### Study period and location

The experiment was carried out from March to May 2018 in the experimental poultry house of the broiler poultry farm of Kuzbass Broiler LLC.

### Experiment design

The animals used were broiler chickens of the ISA F15 cross from 1 to 40 days of age. One control and five experimental groups of day-old broiler chickens were formed, taking into account sex and body weight, with each group consisting of 50 birds. During the experiment, chickens were kept in a floor-managed poultry house with controlled microclimate parameters recommended for the poultry of this cross. When forming the groups, we considered the provisions in the “methods for conducting scientific and industrial research on feeding poultry” [11]. The research scheme provides for the daily feeding of the control group chickens with full compound feed according to the growing phases. The broilers of the experimental groups were given additional feeding of various doses of milk thistle extract: The first experimental group received 0.1 mg/kg body weight, the second experimental group received 0.5 mg/kg of body weight, the third experimental group received 1.0 mg/kg of body weight, the fourth experimental group received 1.5 mg/kg of body weight, and the fifth experimental group received 2.0 mg/kg of body weight. The extract was introduced into the compound feed by three-stage mixing, and the feeding rate was calculated according to the results of the weekly weighing of chickens. The compound feed was fed *ad libitum*.

### Supplement technology

Milk thistle extract was obtained from the Agroecology Research Laboratory of the Kuzbass State Agricultural Academy, Russia, using water-ethanol extraction followed by low-temperature vacuum drying. The extract contains biologically active compounds in quantities that meet the requirements of regulatory documents [12].

### Doses and duration of supplement administration

Doses of the extract introduced into the broiler chickens ration were calculated based on the main biologically active substances under the recommendations of Tutelyan *et al*. [13]. The experiment lasted 40 days. The supplement was excluded from the diet of the poultry 7 days before slaughter.

### Methods for conducting hematological and biological studies

To study the effect of feeding milk thistle extract on the physiological status of the bird, blood samples were collected from the wing vein in six chickens from each group (three males and three females) at the end of the feeding period to determine the hematological and biochemical parameters of the blood. General blood analysis (the number of red blood cells, hemoglobin, and white blood cells) was conducted on an automatic hematology analyzer using a Siemens Advia 2120 (Siemens Healthcare GmbH, Germany) laser unit with the impedance method to detect the diffraction pattern. Blood biochemical parameters (characterizing protein, carbohydrate, lipid, and mineral metabolism) were determined on an automatic biochemical analyzer, Abbott Architect c8000 (Abbott Laboratories, USA). Total protein was determined using photometric biuret method, glucose-hexokinase kinetic method, triglycerides-enzymatic photometric method, alanine aminotransferase (ALT) and aspartate aminotransferase (AST)-kinetic photometric method, calcium-photometry with arsenazo-III complex one, and phosphorus-molybdate photometric method. Protein fractions were determined by the electrophoretic method on cellulose acetate films using a UEF-01 Astra device (OOO NPC Astra, Russia). All methods were unified and recommended by the International Federation of Clinical Chemistry. External quality control was provided by the Federal Service of External Evaluation for Quality of Laboratory Tests and External Quality Assurance Service programs.

### Statistical analysis

Statistical analysis of the obtained data was carried out using the IBM SPSS Statistics Version 22 software (IBM Corp, USA), calculating the mean (M), standard deviation (s), and standard error of deviation (m). The significance of differences between the control and each of the experimental groups was determined using Student’s *t*-test. p<0.05 was considered statistically significant.

## Results

As a result of feeding various doses of milk thistle extract, the number of red blood cells in the blood of broiler chickens from the experimental groups changed (Table-1). Thus, at the end of the experiment, an increase in the number of red blood cells (p>0.05) was found in the blood of broiler chickens in all experimental groups compared to the control group: In the first experimental group by 1.5%, the second and third groups by 6.3%, the fourth group by 8.5%, and in the fifth experimental group by 11.1%.

**Table-1:** Morphological parameters of the blood of broiler chickens.

Parameter	Group					

	Control	1^st^ experimental	2^nd^ experimental	3^rd^ experimental	4^th^ experimental	5^th^ experimental
Red blood cells, 10^12^/L	2.70±0.09	2.74±0.07	2.87±0.04	2.87±0.06	2.93±0.09	3.00±0.12
Hemoglobin, g/L	91.00±4.00	91.16±2.5	93.80±1.75	95.80±1.87	97.22±2.67	99.39±3.84
White blood cells, 10^9^/L	24.70±4.43	25.15±3.94	25.60±2.4	27.36±1.56	27.09±3.17	27.78±5.24

*p<0.05

The hemoglobin content in the blood of broiler chickens of the experimental groups at the end of the study was higher compared to the control group by 0.2%, 3.1%, 5.3%, 6.8%, and 9.2%, respectively (p>0.05 in all cases) (Table-1).In the blood of the chickens of the experimental groups, the number of white blood cells increased (p>0.05) compared to the control group by 1.8%, 3.6%, 10.8%, 9.7%, and 12.5%, respectively (Table-1).

The inclusion of milk thistle extract in the diet of chickens of the experimental groups contributed to a decrease in the total protein content to 34.17-35.43 g/L, which is 0.7-4.2% lower (p<0.05) than the control group (Table-2). The content of albumin in the blood serum of chickens from the experimental groups was lower compared to the control group by 2.5%, 8.6%, 4.9%, 9.4%, and 11.8% (p<0.05 in all cases), respectively (Table-2). The content of α-globulins in the blood serum of chickens of the experimental groups was slightly lower than that of the control group, respectively, by 0.4%, 1.3%, 0.4%, 1.0%, and 1.2% (p<0.05 in all cases) (Table-2).

**Table-2:** Biochemical parameters of the blood of broiler chickens, characterizing protein metabolism.

Parameter	Group					

	Control	1^st^ experimental	2^nd^ experimental	3^rd^ experimental	4^th^ experimental	5^th^ experimental
Total protein, g/L	35.67±0.37	35.43±0.47	34.83±1.04	35.00±1.20	34.50±1.56	34.17±2.17
Protein fractions, %: Albumins	60.68±2.59	58.15±2.43	52.12±7.97	55.78±2.80	51.29±6.64	48.84±9.08
α-Globulins	13.38±0.53	12.97±0.45	12.05±0.45	12.95±0.69	12.36±0.69	12.14±0.89
β-Globulins	15.83±0.41	15.61±0.51	15.20±0.87	15.95±0.84	15.78±1.20	15.84±1.77
γ-Globulins	10.10±2.42	13.26±2.80	20.63±8.23	15.32±1.92	20.57±5.44	23.18±6.98
ALT, U/L	8.00±1.00	10.67±3.44	9.83±3.50	7.17±1.50	8.33±3.00	10.67±2.78
AST, U/L	148.67±17.89	144.67±28.78	151.33±15.22	156.83±11.83	178.33±10.56	147.17±17.11

*p<0.05. ALT=Alanine aminotransferase, AST=Aspartate aminotransferase

In broiler chickens of the first, second, and fourth experimental groups, the content of b-globulins in the blood serum was lower than the control group by 0.2%, 0.6%, and 0.05% ([p<0.05] in all cases), respectively, and that of the third and fifth experimental groups was higher by 0.12% (p>0.05) and 0.01% (p>0.05), respectively (Table-2). When analyzing the content of γ-globulins in the blood serum of broiler chickens of the experimental groups, there was a tendency to their increase compared to the control group by 3.2%, 10.5%, 5.2%, 10.5%, and 13.1% ([p>0.05] in all cases), respectively (Table-2).

The ALT concentration (Table-2) in the blood serum of broiler chickens in the third experimental group was 10.4% lower (p>0.05) than that in the control group. At the end of the study, an increase in the values of the parameter within the limits of average values for broiler chickens had occurred in the blood serum of the first, second, fourth, and fifth experimental groups by 33.4%, 22.9%, 4.1%, and 33.4% ([p>0.05] in all cases), respectively, compared to the control group. Analysis of the AST content in the blood serum of the broiler chicken showed an increase in the second, third, and fourth experimental groups by 1.8%, 5.5%, and 20.0% ([p>0.05] in all cases), respectively, and a decrease in the first and fifth experimental groups by 2.7% and 1.0% ([p<0.05] in all cases), respectively, compared to the control group. We observed 3.2% increase (p>0.05) in the content of triglycerides (Table-3) in the blood serum of the birds of the second and fourth experimental groups and a decrease in their content in the first, third, and fifth experimental groups by 6.3%, 15.2%, and 5.1% ([p<0.05] in all cases), respectively, compared with the control group.

**Table-3:** Biochemical parameters of the blood of broiler chickens, characterizing carbohydrate, lipid and mineral metabolism.

Parameter (mmol/l)	Group					

	Control	1^st^ experimental	2^nd^ experimental	3^rd^ experimental	4^th^ experimental	5^th^ experimental
Triglycerides	1.58±0.15	1.48±0.06	1.63±0.10	1.34±0.32	1.63±0.11	1.50±0.03
Glucose	12.48±2.55	10.47±0.64	10.68±0.08	11.43±1.72	10.93±1.19	11.62±1.79
Calcium	3.32±0.56	2.58±0.22	3.18±0.68	3.20±0.70	2.80±0.53	2.60±0.30
Phosphorus	1.70±0.98	1.67±0.36	3.05±1.63	2.70±0.87	3.07±1.57	1.81±0.80

*p<0.05

When using milk thistle extract in feeding chickens of the experimental groups in the blood serum, a decrease in glucose concentration was found by 16.1%, 14.4%, 8.4%, 12.4%, and 6.9% ([p<0.05] in all cases), respectively, compared to the control group, which may indicate its active use by the bird’s tissues (Table-3). At the end of the experiment, a decrease in the level of calcium in the blood serum of broiler chickens of all five experimental groups was found compared to the control group by 22.3%, 4.2%, 3.6%, 15.7%, and 21.7%, respectively. The differences compared with the control were not statistically significant (p<0.05) (Table-3).

The content of inorganic phosphorus in the blood serum of broiler chickens of the second, third, fourth, and fifth experimental groups at the end of the research was higher than that of the control group by 79.4%, 58.8%, 80.6%, and 6.5% ([p>0.05] in all cases), respectively, and in the first experimental group, it was lower by 1.8% (p<0.05) than that of the control group.

## Discussion

The analysis of blood hematological and biochemical parameters of broiler chickens made it possible to assess the functional state of their body, liver, and other organs, as well as the protein, carbohydrate, and fat metabolism. The concentration of total protein in serum depends mainly on the synthesis and breakdown of two main protein fractions, albumin and globulins. The study of individual protein fractions is important for the diagnosis of pathologies in which the total protein content in the blood serum does not change significantly.

As a result of the studies, we found that by feeding the milk thistle extract, there was a slight decrease in the content of total protein, albumin, α-globulins, and b-globulins in the blood serum of broiler chickens. This might be due to the increased anabolic processes in the birds’ bodies. The increase in the content of γ-globulins in the blood serum of chickens from the experimental groups compared to the control group is a positive trend, which is probably associated with the activation of immune processes and an increase in the resistance of the poultry organism.

An important marker of liver function is the level of activity of AST and ALT, the enzymes that are important links in protein metabolism in the body that catalyzes the transfer of amino groups between amino acids. The observed changes in the content of AST in the blood serum of broiler chickens when feeding milk thistle extract may indicate changes in the processes of transamination and restructuring of their metabolic status.

Blood glucose is the main parameter of carbohydrate metabolism, which reflects the relationship between the processes of its formation and use in tissues. A decrease in the content of glucose in the blood serum of broiler chickens of the experimental groups was established, which may indicate its active use by bird’s tissues. In the blood serum of broiler chickens of the second, third, fourth, and fifth experimental groups fed with milk thistle extract at doses of 0.5, 1.0, 1.5, and 2.0 mg/kg of body weight, level of calcium decreased by 4.2%-22.3% (p<0.05), and the content of phosphorus increased by 6.5%-80.6% (p>0.05), which may indicate the active use of these macroelements in the skeleton formation of an intensively growing bird. The blood indices of analogous chickens from the control and experimental groups corresponded to the average values of the indices for this species and the cross of poultry [14].

Thus, the experiment results showed that the use of milk thistle extract in feeding broiler chickens in doses of 0.1, 0.5, 1.0, 1.5, and 2.0 mg/kg of body weight had a positive effect on erythropoiesis, hemoglobin synthesis, and leukopoiesis, which is confirmed by the increased content of red blood cells, hemoglobin, and white blood cells in the blood of broiler chickens of the experimental groups.

A positive tendency was established with increasing the content of γ-globulins in the blood serum of chickens when feeding various doses of milk thistle extract by 3.2-13.1% (p>0.05) compared to the control group. Improvement of the functional state of the immune system in poultry when feeding milk thistle fruits was noted in the works published by other authors [15,16]. In previous publications [16,17], authors mention a decrease in parameters characterizing lipid metabolism when feeding quails and broiler chickens with various doses of powder or oil cake from milk thistle seeds, which was also confirmed in our studies.

Our data are consistent with the results of other scientists [18] who found that milk thistle silymarin normalized blood biochemical parameters.

## Conclusion

The introduction of milk thistle extract into the diet of broiler chickens in the indicated doses led to an increase in the count of red and white blood cells and levels of hemoglobin and to an increase in the content of γ-globulins, and a decrease in the level of albumin in its serum, which can be regarded as evidence of stimulation processes of hematopoiesis, functions of the immune system, and body growth.

## Authors’ Contributions

OB: Conception and design, acquisition of data, analysis and interpretation of data, drafting and revision of the manuscript. SS: Conception and design, analysis and interpretation of data, drafting and revision of the manuscript. AntS: Conception and design. OP: Analysis and interpretation of data and drafting of the manuscript. AnnS: Acquisition of data and analysis and interpretation of data. GV: Acquisition of data. EU: Drafting and revision of the manuscript. All authors read and approved the final manuscript.
